# Synthesis and pharmacological activity of the epimers of hexahydrocannabinol (HHC)

**DOI:** 10.1038/s41598-023-38188-5

**Published:** 2023-07-08

**Authors:** Fabiana Russo, Maria Angela Vandelli, Giuseppe Biagini, Martin Schmid, Livio Luongo, Michela Perrone, Federica Ricciardi, Sabatino Maione, Aldo Laganà, Anna Laura Capriotti, Alfonso Gallo, Luigi Carbone, Elisabetta Perrone, Giuseppe Gigli, Giuseppe Cannazza, Cinzia Citti

**Affiliations:** 1 Clinical and Experimental Medicine PhD Program, University of Modena and Reggio Emilia, 41125 Modena, Italy; 2 Department of Biomedical, Metabolic and Neural Sciences, University of Modena and Reggio Emilia, 41125 Modena, Italy; 3 Department of Life Sciences, University of Modena and Reggio Emilia, Via Campi 103, 41125 Modena, Italy; 4 Department of Pharmaceutical Chemistry, Institute of Pharmaceutical Sciences, University of Graz, Schubertstraße 1, 8010 Graz, Austria; 5 Division of Pharmacology, Department of Experimental Medicine, Università Della Campania “L. Vanvitelli”, Via Santa Maria di Costantinopoli 16, 80138 Naples, Italy; 6 Department of Chemistry, Sapienza University of Rome, Piazzale Aldo Moro 5, 00185 Rome, Italy; 7 Department of Chemistry, Istituto Zooprofilattico Sperimentale del Mezzogiorno, Via Salute 2, 80055 Portici, Italy; 8 Institute of Nanotechnology – CNR NANOTEC, Campus Ecotekne, Via Monteroni, 73100 Lecce, Italy

**Keywords:** Behavioural methods, Isolation, separation and purification, Mass spectrometry

## Abstract

Cannabis is a multifaceted plant with numerous therapeutic properties on one hand, and controversial psychotropic activities on the other hand, which are modulated by CB1 endocannabinoid receptors. Δ9-Tetrahydrocannabinol (Δ9-THC) has been identified as the main component responsible for the psychotropic effects, while its constitutional isomer cannabidiol (CBD) has shown completely different pharmacological properties. Due to its reported beneficial effects, Cannabis has gained global popularity and is openly sold in shops and online. To circumvent legal restrictions, semi-synthetic derivatives of CBD are now frequently added to cannabis products, producing "high" effects similar to those induced by Δ9-THC. The first semi-synthetic cannabinoid to appear in the EU was obtained through cyclization and hydrogenation of CBD, and is known as hexahydrocannabinol (HHC). Currently, there is limited knowledge regarding HHC, its pharmacological properties, and its prevalence, as it is not commonly investigated in routine toxicological assays. In this study, synthetic strategies were explored to obtain an excess of the active epimer of HHC. Furthermore, the two epimers were purified and individually tested for their cannabinomimetic activity. Lastly, a simple and rapid chromatographic method employing a UV detector and a high-resolution mass spectrometer was applied to identify and quantify up to ten major phytocannabinoids, as well as the HHC epimers, in commercial cannabis samples.

## Introduction

Cannabis is the most widely used illicit drug, with an estimated 4% of the population (15–64 years old) in 2019 reported to have used it, according to the World Drug Report 2021^1^. Δ^9^-Tetrahydrocannabinol (Δ^9^-THC), which was identified by Raphael Mechoulam in the early 1960s, is the primary phytocannabinoid responsible for the psychotropic effects of cannabis. The constitutional isomer of Δ^9^-THC, cannabidiol (CBD), is the second most abundant phytocannabinoid found in cannabis and does not exhibit any narcotic activity. CBD is predominantly present in cannabis cultivated for industrial purposes, commonly referred to as hemp varieties. Other so-called “minor” phytocannabinoids have been isolated and characterized, and some of them have demonstrated euphoriant activity similar to Δ^9^-THC. Among the recently discovered THC-type phytocannabinoids, such as Δ^9^-tetrahydrocannabiphorol (Δ^9^-THCP)^2^, Δ^9^-tetrahydrocannabihexol (Δ^9^-THCH)^3^, and Δ^9^-tetrahydrocannabibutol (Δ^9^-THCB)^4^, the heptyl homologue has exhibited an unequivocal cannabinomimetic activity even higher than Δ^9^-THC in animal experiments^2^.

After the identification of Δ^9^-THC as the main psychotropic component of cannabis, chemical and pharmaceutical research focused on modifying the lead compound to obtain more potent analogues^5^. Additionally, the discovery of the endocannabinoid receptors CB1 and CB2 has led to the identification of new scaffolds, enabling the development of increasingly potent synthetic cannabinoids (SCs). However, such compounds, which have limited therapeutic utility, are generally synthesized in illegal laboratories and sold for recreational use. Recently, over 250 synthetic compounds targeting the endocannabinoid system have been developed in clandestine laboratories^6^. These compounds are typically added to products often referred to as “Spice” or “K2” and distributed to end-users^7^. In contrast to THC, which has a relatively large safety margin when used recreationally and requires higher doses to cause serious adverse effects^8^, the use of SCs has been documented as completely unsafe, with a range of dangerous side effects^9^.

Low-THC cannabis varieties (containing up to 0.3% THC) have recently been legalized in the USA^10^. These varieties can have high levels of CBD, which lacks cannabinomimetic properties and has been recognized as a potent antiepileptic drug^11^. However, when CBD is treated with acids, it cyclizes to form Δ^9^-THC and Δ^8^-THC^12^. Both THC isomers are subject to international restrictions and are scheduled under Schedule II of the United Nations Convention on Psychotropic Substances^13^. To address the marketing of controlled substances, the development of semi-synthetic derivatives of CBD has become a widely employed strategy in the USA. The first semi-synthetic cannabinoid (SSC) reported in the EU and monitored as a novel psychoactive substance (NPS) by the EU Early Warning System since October 21, 2022, is hexahydrocannabinol, better known as HHC^14^. HHC (hexahydrocannabinol) was first described in 1940 by Adams^15^, but it has only recently gained attention from toxicologists and analysts^16^. Despite its long history, few experimental studies have investigated its effects, particularly in humans. HHC is often sold as a “legal” alternative to illegal THC, and it is commonly sprayed onto or mixed with cannabis products marketed as “legal highs”^14^.

HHC has three stereogenic centres, which theoretically give rise to eight stereoisomers. However, in practice, HHC is consistently found as two epimers: 9*R* and 9*S*, while the configuration at C10*a* and C6*a* remains unchanged. Early studies on HHC’s biological activity in animals, although compromised by the low purity of the material used, indicated that HHC exhibited lower marijuana-like activity compared to THC^17,18^. Subsequent studies using better-characterized material revealed that the R epimer is the one responsible for cannabimimetic activity, while the S epimer is either devoid of or has less psychotropic effect^19^.

The psychotropic activity of HHC epimers was evaluated in a study by Mechoulam et al. in 1980, which involved administering the individual epimers of HHC to rhesus monkeys^20^. The study found that the epimer with the equatorial methyl substituent ((*R*)-HHC) induced severe stupor, ataxia, immobility, and other effects indicative of high potency, even at low doses. The epimer with the axial methyl group ((*S*)-HHC) induced drowsiness and decreased motor activity at higher doses^20^. However, the authors noted that the compound used was not pure, and impurities of the other epimer could have influenced the results^20^.

**Figure 1:**
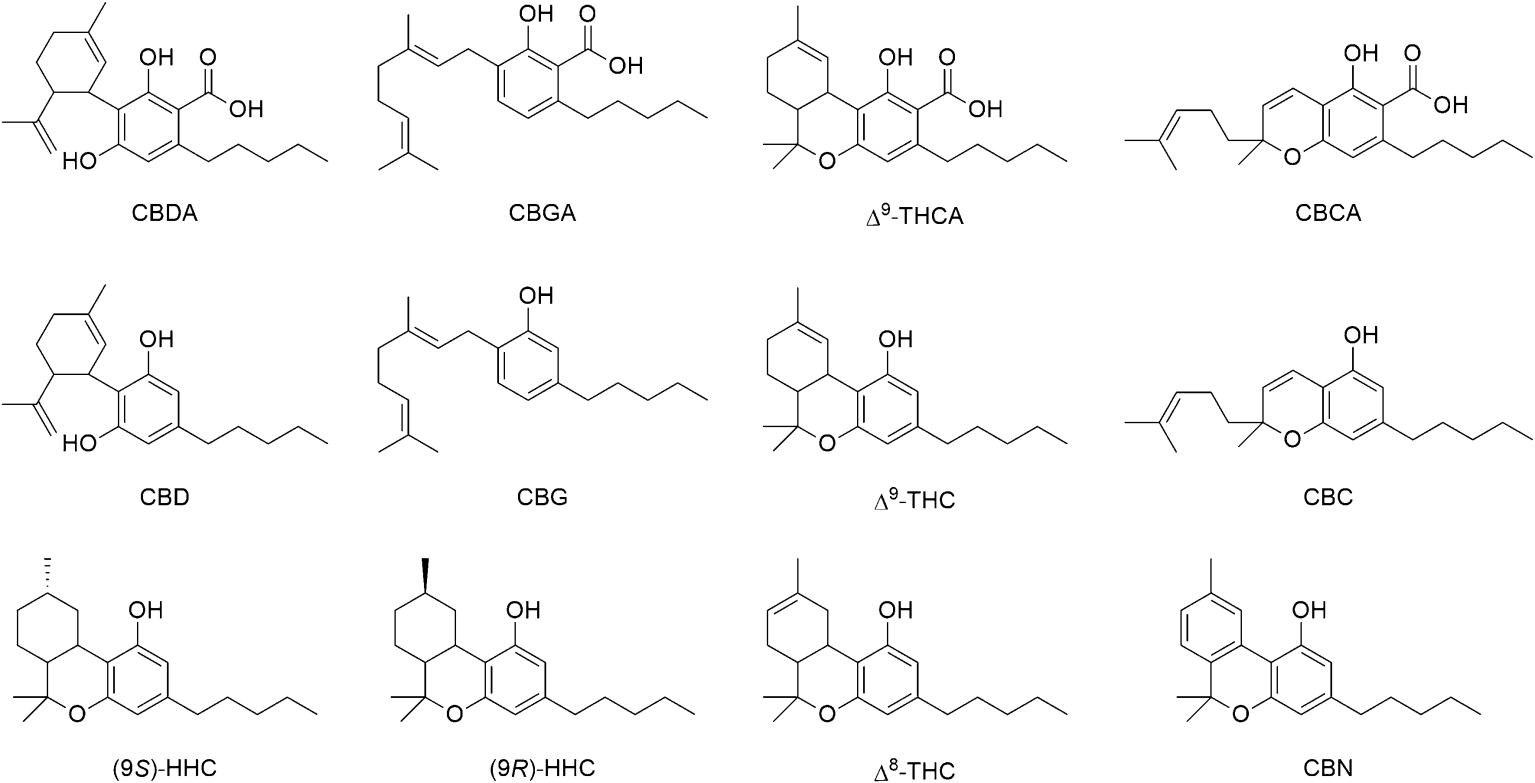
Chemical structure of the analyzed cannabinoids.

The potency of a cannabis product containing HHC depends on the abundance of one epimer relative to the other. Recent studies have reported an excess of the 9*R* epimer in certain industrial hemp products^16^. Therefore, it is important to investigate the formation of one epimer over the other starting from CBD and analyze the epimeric ratio in HHC-containing cannabis products. The present study aims to evaluate the epimeric excess resulting from different synthetic strategies and determine the concentrations of both epimers in commercial HHC-containing cannabis products. To achieve these goals, high-performance liquid chromatography coupled with a diode array detector and high-resolution mass spectrometry (HPLC–DAD-HRMS) was employed. In addition to HHC epimers, ten other phytocannabinoids commonly found in cannabis extracts were analyzed, including CBD, Δ^9^-THC, Δ^8^-THC, CBG, CBC, their corresponding carboxylated native precursors (cannabidiolic acid (CBDA), tetrahydrocannabinolic acid (THCA), cannabigerolic acid (CBGA), and cannabichromenic acid (CBCA)), and the THC oxidation product cannabinol (CBN) (Fig. 1). Lastly, the tetrad test, a behavioral assay, was performed on mice after administering the individual HHC epimers, and the results were compared to those reported for Δ^9^-THC. This test helps evaluate the psychoactive effects of substances.

## Results

### Synthesis of HHC epimers

The synthesis of HHC epimers was carried out by subjecting CBD to acidic treatment. Depending on the reaction conditions, cyclization and hydrogenation of CBD can lead to the formation of either Δ^9^-THC or Δ^8^-THC as reaction intermediates. Gaoni and Mechoulam, in particular, obtained Δ^9^-THC by treating CBD with HCl as a catalyst for a short time (2 h), while Δ^8^-THC was obtained when CBD was treated with *p*-toluensulfonic acid (*p*TSA) for a longer duration (18 h)^21^. Subsequent reduction of the double bond on the terpene group resulted in a mixture of the two HHC epimers. Hydrogenation of Δ^9^-THC yielded an excess of the *S* epimer compared to the *R* epimer (approximately 2:1 ratio), whereas hydrogenation of Δ^8^-THC gave a 3:1 epimeric ratio in favour of the *R* epimer^21^.

**Figure 2:**
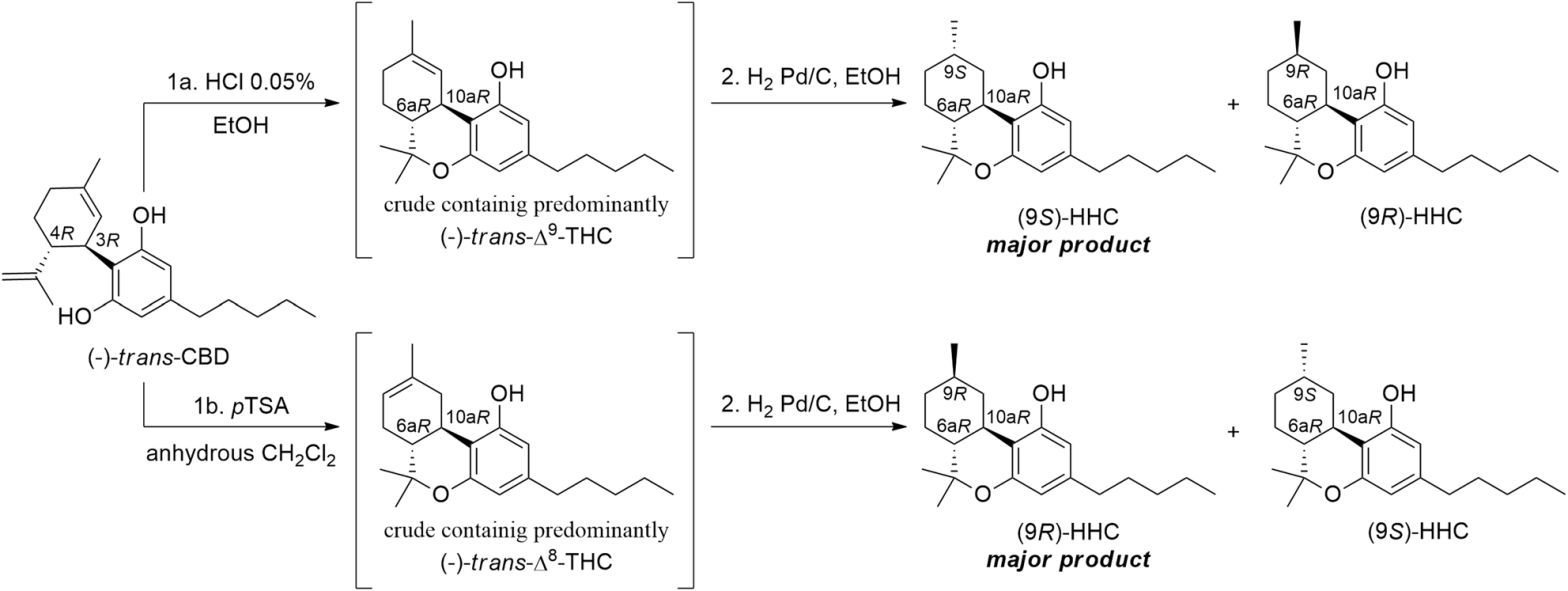
Synthesis of HHC. Step 1a-b: Cyclization of CBD. Step 2: Hydrogenation of the crude reaction mixture.

Based on this knowledge, CBD was treated with either HCl or *p*TSA for specific durations, followed by hydrogenation without purification of the intermediates. HHC was obtained as the product in both reactions, but with different epimeric ratios depending on the reaction conditions. Specifically, the reaction with HCl for 2 h resulted in a 57:43 *S*/*R* ratio, while the reaction with *p*TSA for 18 h yielded a 61:39 *R*/*S* ratio (Fig. 2).

**Figure 3:**
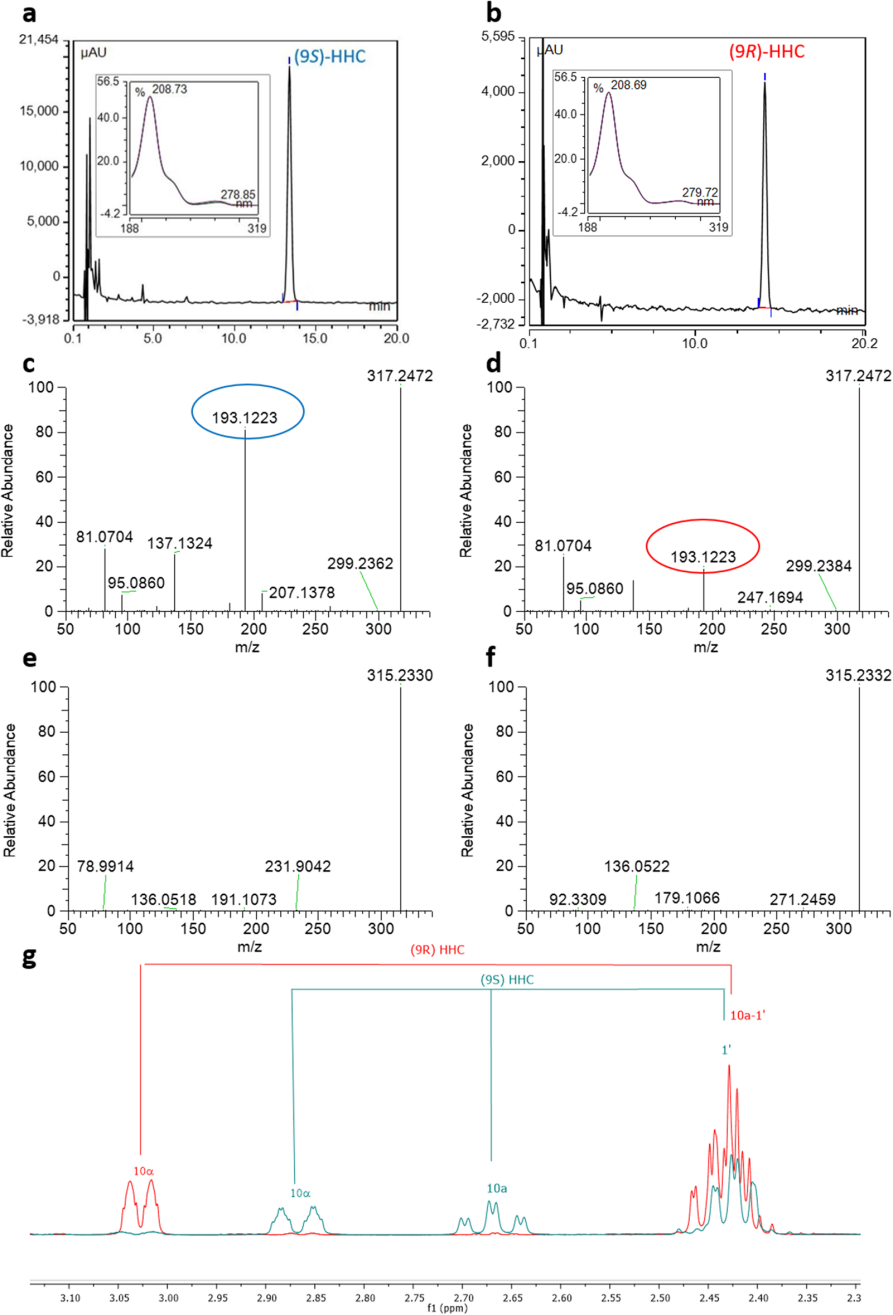
HPLC–UV-HRMS/MS and NMR characterization of the isolated (9*S*)-HHC and (9*R*)-HHC. HPLC–UV trace of (9*S*)-HHC (**a**) and (9*R*)-HHC (**b**) with the respective UV spectrum in the boxes; HRMS/MS pattern in HESI + mode of (9*S*)-HHC (**c**) and (9*R*)-HHC (**d**) (the discriminant fragment is circled in blue and red for (9*S*)-HHC and (9*R*)-HHC respectively); HRMS/MS pattern in HESI- mode of (9*S*)-HHC (**e**) and (9*R*)-HHC (**f**); discriminant chemical shifts in the ^1^H NMR spectra of (9*S*)-HHC (blue) and (9*R*)-HHC (red) (**g**).

The individual HHC epimers, (9*S*)-HHC and (9*R*)-HHC, were isolated using semi-preparative HPLC, achieving purity grades exceeding 95% and 99%, respectively, which were sufficient for conducting the biological tests. UV, MS, and NMR analyses were performed to characterize both epimers. The UV spectra were found to be superimposable (Fig. 3a, b), while the MS^2^ dimension showed similar patterns, distinguished only by the relative abundance (RA) of a fragment at *m/z* 193.1223 in HESI + mode. This fragment likely corresponds to the resorcinol group attached to one carbon atom of the reduced terpene moiety, with the oxygen atom no longer included in the pyran ring (Fig. 3c, d). In the mass fragmentation spectrum, the *S* epimer had an RA of 83% for this fragment, whereas the *R* epimer had an RA of 20%. In contrast, the HESI- mode produced identical fragmentation patterns (Fig. 3e, f). Furthermore, the ^1^H NMR analysis revealed distinctive differences, indicating that the first eluted epimer on reversed-phase HPLC corresponds to (9*S*)-HHC, while the second eluted epimer corresponds to (9*R*)-HHC (Fig. 3g). The stereochemistry at C9 was determined by comparing the NMR data obtained in this study with those reported in the literature by Archer et al.^22^ and Gaoni et al.^21^. Archer et al. provided a partial spectrum of a (*S*)-HHC/(*R*)-HHC mixture with a 3:1 ratio, demonstrating that the signals indicated as “major” corresponded to the *S* isomer, while those indicated as “minor” corresponded to the *R* isomer^22^. The findings of Gaoni et al. and Archer's work support the characterization of the HHC epimers based on the NMR spectra. Gaoni et al. suggested that the isomer labeled as VIa, which exhibited a proton signal at C10*a* (C3 in the old numbering system) appearing “as a broad doublet centered at 2.85 ppm”, corresponded to the isomer with the methyl group at C9 (C1 in the old numbering system) in the axial position ((*S*)-HHC). Conversely, the “broad doublet centered at 3.05 ppm” in structure VIb corresponded to the isomer with the methyl group at C9 in the equatorial position ((*R*)-HHC)^21^. In the current study, a peculiar shift in the NMR spectrum was observed for the hydrogen attached to C10α, with a change from 2.85 ppm in (*S*)-HHC to 3.03 ppm in (*R*)-HHC. Additionally, the hydrogen atom of C10*a* exhibited a shift from 2.67 ppm in the *S* epimer to 2.49–2.37 ppm in the *R* epimer. These observed shifts are consistent with the data reported in the previously mentioned works^21,22^, further confirming the assignment of the stereochemistry of the HHC epimers in this study.

### HPLC–UV-HRMS analysis

**Figure 4:**
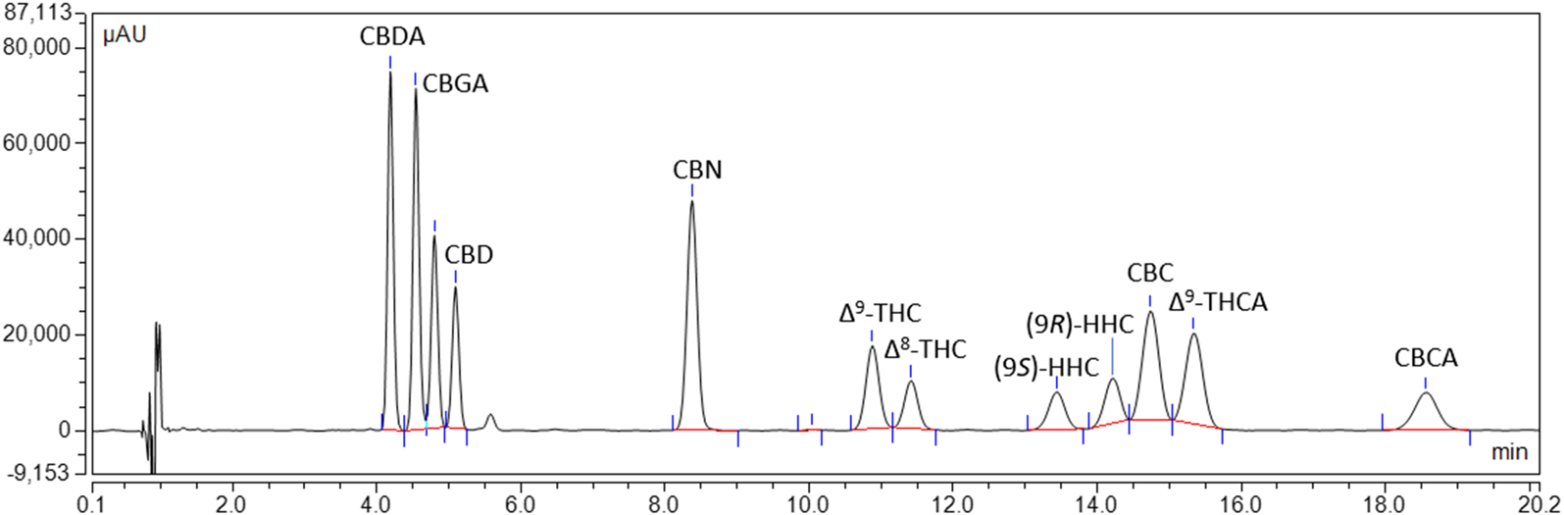
HPLC–UV chromatogram of a cannabinoid standard mixture. HPLC–UV chromatogram of a standard mixture containing ten phytocannabinoids (CBDA, CBGA, CBG, CBD, CBN, Δ^9^-THC, Δ^8^-THC, CBC, THCA, and CBCA) and the two HHC epimers at the concentration of 10 µg/mL.

A previously reported and validated HPLC–UV-HRMS method^23^ was adapted for the determination of ten phytocannabinoids and the HHC epimers. A C18 stationary phase with core–shell technology was utilized for the separation of all phytocannabinoids, and an isocratic elution program with 70% ACN for 20 min provided good baseline resolution of all peaks. Figure 4 displays the UV traces of all cannabinoid standards at a concentration of 10 µg/mL at 228 nm.

The method was applied to analyze all phytocannabinoids and HHC epimers in ethanolic extracts of two hemp biomass samples (HHC-1 and HHC-2), one HHC hashish sample (HHC-3), and a pure HHC sample (HHC-4) (Figure S5, Supporting Information). Extraction of the analytes was performed using ethanol, as previous studies have demonstrated its superior ability to extract this class of compounds^2–4,24–26^. The chromatograms of the extracts were examined for interfering compounds using HRMS, which confirmed the absence of other cannabinoids at the retention time of the analytes. All extracts were found to contain both epimers, with the *R* form being approximately twice as abundant as the *S* form. In the sample claimed to be pure HHC, the two epimers were present in a 58:42 mixture. In the three analyzed extracts, the amount of the *S* epimer ranged from 8.40 to 11.66% (on dry weight), while the *R* epimer was found in the range of 17.13% to 26.46%.

All extracts exhibited a prominent presence of CBDA and CBD, along with lower amounts of CBGA, indicating that the inflorescence originated from industrial hemp varieties. Specifically, CBDA ranged from 2.8 to 7.18%, and CBD ranged from 1.20 to 2.60%. CBGA was also detected in low concentrations, ranging from 0.06 to 0.26%. Additionally, CBN was consistently present in all samples, with concentrations ranging from 0.05 to 0.21%. All samples showed the presence of both Δ^9^-THC and Δ^8^-THC, with the former being predominant. On the other hand, THCA and CBCA were present in trace amounts. The cannabinoid concentrations in the analyzed samples are reported in Table 1.

**Table 1:** Concentration of phytocannabinoids and HHC epimers in the three extracts and in the commercial HHC mixture. Values are expressed in % (w/w) as mean ± SD (n = 3).

	HHC-1	HHC-2	HHC-3	HHC-4
CBDA	7.54 ± 0.51	4.38 ± 0.26	2.91 ± 0.13	< LOD
CBGA	0.12 ± 0.03	0.29 ± 0.04	0.11 ± 0.07	< LOD
CBG	< LOQ	< LOQ	< LOQ	< LOD
CBD	1.25 ± 0.07	1.51 ± 0.12	2.83 ± 0.33	< LOD
CBN	< LOQ	< LOQ	0.22 ± 0.01	0.13
Δ^9^-THC	0.35 ± 0.02	0.26 ± 0.01	1.97 ± 0.12	0.29 ± 0.00
Δ^8^-THC	0.12 ± 0.04	< LOD	0.24 ± 0.01	0.16 ± 0.03
CBC	< LOD	< LOD	< LOD	< LOD
(9*S*)-HHC	9.36 ± 0.43	8.78 ± 0.54	11.61 ± 0.08	4.15 ± 0.07
(9*R*)-HHC	18.71 ± 0.97	17.73 ± 0.86	26.50 ± 0.07	5.60 ± 0.28
Δ^9^-THCA	0.15 ± 0.05	< LOQ	< LOD	< LOD
CBCA	< LOD	< LOD	< LOD	< LOD

Isomers of HHC could be tentatively identified in all extracts by HRMS, along with other oxidation derivatives of HHC. Since no standard was available for these compounds, only the molecular formula could be hypothesized (Figure S6). Interestingly, the sample of pure HHC exhibited a unique peak at 12.96 min with a precursor ion [M + H] + at *m/z* 319.2629 and [M-H]- at *m/z* 317.2488, displaying higher intensity in positive ionization mode (Figure S6). This compound, with a predicted molecular formula of C_21_H_34_O_2_, presented a characteristic fragment at *m/z* 139.1480, which differed by two hydrogen atoms from that of HHC (137.1324) and by four hydrogen atoms from CBD- and THC-like cannabinoids (135.1174). This fragment could likely be attributed to the terpene moiety. Based on the chromatographic data, which showed a longer retention time compared to CBD, and the HRMS fragmentation pattern, a CBD-like structure with all saturated C–C bonds on the terpene moiety could be proposed. As its retention time fell between THC and HHC species, the presence of an additional free hydroxyl group could explain its higher hydrophilicity compared to HHC, while the complete saturation of the terpene moiety could account for its higher lipophilicity compared to THC. This compound is likely to derive from the hydrogenation of residual CBD starting material in the reaction mixture, known as H4CBD. The spectroscopic data aligns with the findings reported by Collins et al., who synthesized and characterized both HHC and H4CBD.

### In vivo determination of the cannabinoid profile of (9*R*)-HHC and (9*S*)-HHC

**Figure 5:**
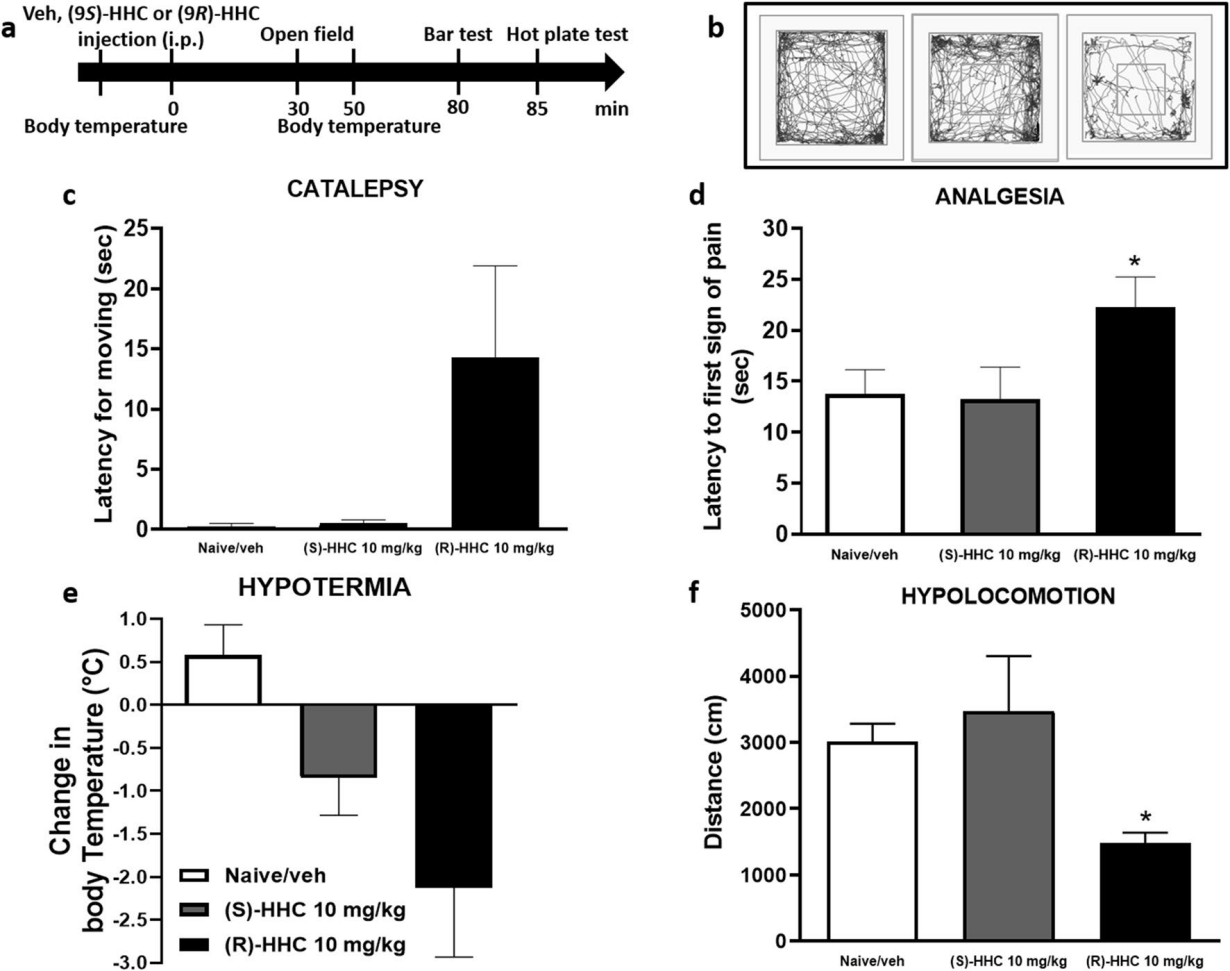
Dose-dependent effects of (9*R*)-HHC and (9*S*)-HHC administration (10 mg/kg, i.p.) on the tetrad behavioural tests in mice in comparison to vehicle. Time schedule of the tetrad tests in min from (9*R*)-HHC, (9*S*)-HHC or vehicle administration (**a**). Locomotion decrease induced by (9*R*)-HHC administration in the open field test (**b**, **f**). Decrease of body temperature after (9*R*)-HHC administration (**e**); the values are expressed as the difference between the basal temperature (i.e., taken before (9*R*)-HHC or vehicle administration) and the temperature measured after (9*R*)-HHC or vehicle administration. Increase in the latency for moving from the catalepsy bar after (9*R*)-HHC administration (**c**). Increase in the latency after the first sign of pain shown by the mouse in the hot plate test following (9*R*)-HHC administration (**d**). Data are represented as the mean ± SEM of 4 mice per group. * indicates significant differences compared to vehicle injection, *p < 0.05 Tukey’s test.

The cannabimimetic activity of (9*R*)-HHC and (9*S*)-HHC was evaluated using a tetrad of behavioral tests on mice. These tests assess spontaneous activity, catalepsy (immobility index), analgesia, and changes in rectal temperature (Fig. 5a), which are physiological manifestations of cannabinoid activity^22^.

After intraperitoneal administration, (9*R*)-HHC at a dose of 10 mg/kg significantly reduced the spontaneous activity of mice in the open field test compared to the vehicle-treated group. The distance covered by mice in the open field was significantly decreased by (9*R*)-HHC (1476.750 ± 159.842 cm, *p* = 0.0183), whereas (9*S*)-HHC did not affect locomotion (3469.750 ± 833.532 cm, *p* = 0.7392) (Figs. 5b and 5f).

In the catalepsy test, (9*R*)-HHC administration increased the latency of moving from the catalepsy bar, indicating a decrease in cataleptic behaviour. Although the difference was not statistically significant compared to the vehicle-treated group, there was a trend towards reduced catalepsy (14.250 ± 7.642 s, *p* = 0.3097) for (9*R*)-HHC (Fig. 5c).

In the hot plate test, (9*R*)-HHC demonstrated a significant antinociceptive effect, indicating analgesic properties. The latency to respond to the hot plate stimulus was significantly increased by (9*R*)-HHC (22.200 ± 3.040 s, *p* = 0.0204) compared to the vehicle-treated group (13.768 ± 2.367 s) (Fig. 5d). In contrast, (9*S*)-HHC did not induce catalepsy (0.500 ± 0.289 s, *p* = 0.6259) or analgesia (13.250 ± 3.146 s, *p* = 0.9934).

Lastly, both (9*R*)-HHC and (9*S*)-HHC showed a trend towards decreasing body temperature, indicating hypothermic effects. However, the difference was not statistically significant compared to the vehicle-treated mice. (9*R*)-HHC exhibited a greater trend towards decreasing body temperature (− 2.125 ± 0.808 °C, *p* = 0.1553) compared to (9*S*)-HHC (− 0.850 ± 0.435 °C, *p* = 0.1137), but further analysis is needed to establish statistical significance (Fig. 5e).

These results suggest that (9*R*)-HHC possesses cannabimimetic activity, as evidenced by decreased locomotor activity, decreased catalepsy, increased analgesia, and a trend towards hypothermia. In contrast, (9*S*)-HHC did not significantly affect these behavioural parameters.

## Discussion

Since the discovery of Δ^9^-THC as the main psychoactive compound in cannabis, efforts have been made by medicinal chemists to modify its chemical structure in order to improve its pharmacological properties. However, very few, if any, of these derivatives have successfully reached the market as approved drugs. On the other hand, over the past fifteen years, a large number of synthetic cannabinoids (SCs) or “spice” have emerged as illegal drugs. These SCs are structurally distinct from Δ^9^-THC and have been marketed as alternatives to cannabis.

In recent years, there has been a resurgence in the cultivation of cannabis for industrial purposes, particularly due to the high concentrations of CBD, which is non-psychoactive and possesses various therapeutic properties. CBD is used in medicinal products for the treatment of certain types of childhood epilepsy^11^. The World Health Organization (WHO) has suggested that CBD should not be classified as a scheduled substance, as it lacks the intoxicating properties associated with THC or other SCs^27^.

In some European countries, the recreational use of industrial hemp inflorescence as a substitute for high-THC cannabis has become a widespread phenomenon known as “light cannabis”. However, the legal status of such products remains unclear in European legislation. In parallel, the USA have allowed the legal marketing of all cannabis products with THC levels below 0.3% with the Farm Bill Act in 2018.

Alongside the emergence of SCs, there has been the appearance of semi-synthetic cannabinoids (SSCs) in the United States. These compounds are derived from chemical modifications of cannabinoids extracted from cannabis. One such compound, HHC, is the hydrogenation product of Δ^9^-THC and Δ^8^-THC. By removing the double bond at either the C9 or C8 position, a new stereogenic center is generated, resulting in either the *R* or *S* epimer. Mechoulam had suggested that it is possible to obtain an excess of the *R* epimer from Δ^8^-THC and an excess of the *S* epimer from Δ^9^-THC^21^.

In vitro studies have indicated that the *R* epimer has a higher affinity for the CB1 receptor, while the *S* epimer has poor affinity^19^. This suggests that different epimeric mixtures with varying intensities of biological effects can be obtained based on the synthetic procedure.

CBD has been shown to isomerize to either Δ^9^-THC or Δ^8^-THC under acidic conditions, depending on factors such as reaction time, catalyst type, and reaction conditions^2,12,21^. As a result, the production of Δ^8^-THC and Δ^9^-THC as semi-synthetic products from CBD extracted from hemp has increased^14^. However, both Δ^8^-THC and Δ^9^-THC are controlled substances and not legally marketed. Therefore, the subsequent reduction of the double bond to form semi-synthetic HHC has allowed for a legal alternative that offers similar psychoactive effects to THC. HHC is currently being sprayed onto hemp products and openly sold to provide the desired “high” effects of cannabis.

The scientific studies on HHC are limited, highlighting the need for further investigation. The researchers’ work demonstrates that HHC can be obtained from CBD, and the reaction conditions can selectively favour the production of one epimer over the other. Using a validated analytical method, the presence of HHC was identified in two commercial samples of hemp biomass (HHC-1 and HHC-2) and one hemp hashish sample (HHC-3). The concentrations of the *R* epimer were consistently higher than those of the *S* epimer in all samples.

The hemp biomass samples also contained significant concentrations of other phytocannabinoids, particularly CBDA and CBD, indicating that they were derived from industrial hemp. The levels of Δ^9^-THC and Δ^8^-THC in the biomass extracts and pure HHC mixture were below 0.5%, while the HHC hashish sample contained around 2% of these phytocannabinoids. The presence of Δ^9^-THC and Δ^8^-THC could be attributed to residues from the HHC synthesis process, although a natural origin cannot be ruled out. The relatively high levels of HHC compared to CBD and THC in the samples suggest its exogenous origin rather than being naturally occurring^28^.

Furthermore, since the *R* epimer was consistently found in excess of the *S* epimer, it is likely that HHC was synthesized starting from CBD with Δ^8^-THC as the reaction intermediate, rather than using Δ^9^-THC. Analysis of the pure HHC sample (HHC-4) revealed the absence of CBDA and CBD, but the presence of CBN, Δ^8^-THC, and Δ^9^-THC as impurities alongside the main component HHC. The *R* epimer was slightly in excess, indicating a similar synthetic route.

To assess the cannabimimetic activity of HHC, the tetrad test in mice was conducted. Due to the limited availability of the two epimers, only one dose (10 mg/kg) was tested, similar to the dose used for THC in the tetrad test. The results showed that (9*R*)-HHC significantly affected two out of the four behaviours in the tetrad test: hypolocomotion (reduced spontaneous activity) and analgesia (pain relief). These findings suggest a partial CB1-mediated psychotropic effect. However, the potency of HHC in inducing the tetrad effects was lower than that of Δ^9^-THC and Δ^9^-THCP, a recently discovered THC analog.

In summary, a likely origin for the first SSC that appeared in the European market has been proposed in this work, and the biological tests confirmed that the cannabimimetic activity of HHC resides in the *R* epimer, while the S epimer has minimal activity, consistent with the in vitro binding studies on the CB1 receptor. Further evaluation is needed to determine whether HHC should be classified as a scheduled narcotic substance.

## Methods

### Chemicals and reagents

Ethanol 96%, acetonitrile (ACN), and formic acid (FA) were all LC–MS grade and were bought from Sigma Aldrich (USA). Ultrapure water was obtained with a water purification system (Direct-Q 3UV, Merck Millipore, Milan, Italy). Chemicals and solvents employed in the synthetic process were reagent grade and used without further purification. The following abbreviations for common organic solvents were adopted: diethyl ether (Et_2_O); dichloromethane (CH_2_Cl_2_); cyclohexane (CE); chloroform (CDCl_3_); ethanol (EtOH). Stock solutions (1 mg/mL) of certified reference cannabinoid standards of CBDA, Δ^9^-THCA, CBGA, CBCA, CBD, CBG, CBC, CBN, as well as of Δ^9^-THC and Δ^8^-THC (500 µg/mL) were bought from Cayman Chemical (Ann Arbor, Michigan, USA). Stock solutions (1 mg/mL) of CBDH, Δ^9^-THCH, (9*R*)-HHC and (9*S*)-HHC were obtained by properly diluting the pure compounds in-house synthesized.

### Plant material

Samples of hemp biomass (HHC-1 and HHC-2), HHC hashish (HHC-3) and pure HHC were purchased from Baked Bologna (Bologna, Italy). The use of *Cannabis sativa* L. plants with THC levels below 0.5% in the present study complies with the Italian guidelines according to the Law 242/2016 and to the common agricultural policy (art. 38–44) of the European Union Treaty (GUCE 26/10/2012).

### Synthetic procedure *a* for HHC

CBD (0.7 mmol) was dissolved in 20 mL of absolute EtOH containing 0.05% HCl and refluxed for 2 h. The resulting crude was neutralized with a saturated solution of Na_2_CO_3_ and subject to hydrogenation with an H-Cube ThalesNano flow reactor (Budapest, Hungary) according to the following experimental conditions: 3 mm 10% Pd/C cartridge, 30 °C, 20 bar, 1 mL/min. The crude product mixture showed a 57:43 *S*/*R* HHC mixture along with other by-products. The solvent was evaporated and the crude of the reaction was purified with semipreparative HPLC–UV (Coloumn Luna 5 μm C18, 100 Å, 250 × 10 mm-Phenomenex, Bologna, Italy). An isocratic elution was employed with mobile phase 80% ACN (with 0.1% FA) and 20% MilliQ water (with 0.1% FA) at a flow rate of 7.5 mL/min. The UV trace was followed at 228 nm and the compounds of interest were obtained with a purity higher than 95% (12 mg for (9*R*)-HHC and 8 mg for (9*S*)-HHC).

### Synthetic procedure *b* for HHC

To a solution of CBD (0.7 mmol) in 15 mL of anhydrous CH_2_Cl_2_, *p*TSA (10 mg, 10% mol) was added at room temperature, under a nitrogen atmosphere. The reaction was stirred in the same conditions for 48 h. After that, the mixture was diluted with Et_2_O and washed with a saturated solution of NaHCO_3_. The organic layer was collected, washed with brine, dried over anhydrous Na_2_SO_4_ and concentrated. The obtained crude was subject to hydrogenation as described for the synthetic procedure *b* to give a crude product with a 61:39 *R*/*S* HHC mixture The two epimers were purified with semi-preparative HPLC as reported above (11 mg for (9*S*)-HHC and 8 mg for (9*R*)-HHC).

### NMR characterization of HHC

NMR spectra were recorded either on a Bruker 400 (working at 400.134 MHz for ^1^H and 100.62 MHz for ^13^C) or a Bruker 600 spectrometer (working at 600.130 MHz for ^1^H and 150.902 MHz for ^13^C). Monodimensional spectra were acquired with a spectral width of 8278 Hz (for ^1^H NMR) and 23.9 kHz (for ^13^C NMR), a relaxation delay of 1 s and a number of transients of 32 and 1024 for ^1^H NMR and ^13^C NMR, respectively. NMR spectra were acquired in CDCl_3_ and chemical shifts (δ) were registered in ppm with respect to that of the residual solvent (δ = 7.26 ppm for ^1^H and δ = 77.20 ppm for ^13^C); coupling constants are reported in Hz, splitting patterns are expressed as singlet (s), doublet (d), triplet (t), quartet (q), double doublet (dd), quintet (qnt), multiplet (m), broad signal (b).

### (9*S*)-6,6,9-trimethyl-3-pentyl-6a,7,8,9,10,10a-hexahydro-6*H*-benzo[*c*]chromen-1-ol ((9*S*)-HHC)

^1^H NMR (400 MHz, CDCl_3_) δ 6.25 (s, 1H, C4), 6.07 (s, 1H, C2), 4.61 (s, 1H, OH), 2.92–2.79 (m, 1H, C10α), 2.67 (td, *J* = 11.4, 2.9 Hz, 1H, C10a), 2.46–2.38 (m, 2H, C1'), 2.11 (m, 1H, C9), 1.68–1.61 (m, 2H, C8-C7), 1.58–152 (m, 2H, C2'), 1.46–1.44 (m, 1H, C6a) 1.36 (s, 3H, C12), 1.33–1.28 (s, 6H, C4'-C3'-C10β), 1.13 (d, *J* = 7.2 Hz, 3H, C11), 1.09 (s, 3H, C13), 0.88 (t, *J* = 7.0 Hz, 3H, C5'). ^13^C NMR (101 MHz, CDCl_3_) δ 155.38, 154.77, 142.65, 110.63, 110.19, 107.79, 77.03, 50.09, 36.33, 35.59, 32.40, 31.77, 30.76, 29.49, 28.06, 27.76, 27.10, 23.26, 22.73, 19.30, 18.98, 14.20.

### (9*R*)-6,6,9-trimethyl-3-pentyl-6a,7,8,9,10,10a-hexahydro-6*H*-benzo[*c*]chromen-1-ol ((9*R*)-HHC)

^1^H NMR (600 MHz, CDCl_3_) δ 6.25 (s, 1H, C4), 6.08 (s, 1H, C2), 4.64 (s, 1H, OH), 3.03 (d, *J* = 12.9 Hz, 1H, C10α), 2.49–2.37 (m, 3H, C1’-C10a), 1.87–1.80 (m, 2H, C8-1H, C7-1H), 1.67–1.60 (m, 1H, C9), 1.59–1.52 (m, 2H, C 2’), 1.46–1.44 (m, 1H, C6a), 1.36 (s, 3H, C12), 1.34–1.24 (m, 4H, C4’-C3’), 1.16–1.07 (m, 2H, C8-1H, C7-1H), 1.06 (s, 3H, C13) 0.94 (d, *J* = 6.6 Hz, 3H, C11), 0.88 (t, *J* = 7.0 Hz, 3H, C5’), 0.78 (m, 1H, C10β). ^13^C NMR (151 MHz, CDCl_3_) δ 155.15, 154.83, 142.74, 110.44, 110.23, 107.77, 77.14, 49.30, 39.16, 35.71, 35.58, 33.03, 31.75, 30.76, 28.24, 27.94, 22.78, 22.73, 19.22, 14.21.

### HPLC–UV-HRMS analysis

A Vanquish Core system (Thermo Fisher Scientific, Waltham, Massachusetts, USA) with binary pump, vacuum degasser, thermostated autosampler and column compartment, and diode array detector (DAD) was interfaced to an Exploris 120 Orbitrap mass analyzer with a heated electrospray ionization source (HESI). The chromatographic separation was achieved on a Poroshell 120 EC C18 (100 × 3.0 mm, 2.7 µm) (Agilent, Milan, Italy) with an isocratic elution at 70% ACN for 20 min and a washing step at 98% ACN for 3 min. The column was re-equilibrated at 70% ACN for further 3 min for a total run time of 26 min at a constant flow rate of 0.5 mL/min.

The HESI parameters were optimized in previous works for cannabinoids: spray voltage 4200 kV and 3800 kV for HESI + and HESI- mode respectively, sheath gas 70 au, auxiliary gas 5 au, sweep gas 0.5 au, ion transfer tube temperature 390 °C and vaporizer temperature 150°C^26,29^. The mass analyzer was operated in both full scan (FS) and data-dependent acquisition (DDA) mode. In FS mode the resolution was set at 60,000 FWHM (full width at half maximum), the RF lens level at 70%, the maximum injection time 54 ms, the *m/z* range at 150–750, and the isolation window at *m/z* 1.2. In DDA mode the resolution was set at 30,000 FWHM, the maximum injection time at 22 ms, the *m/z* range at 50–750, the isolation window at *m/z* 1.2, and the stepped normalized collision energy (NCE) at 20–40-100^26,29^. The injection volume was 5 µL. The analyses were acquired with Xcalibur 3.0 and processed using Chromeleon 7 for the UV traces and TraceFinder 54.0 for the MS traces (all from Thermo Fisher Scientific).

### Calibration standards and sample preparation and cannabinoids quantification

Calibration solutions of all phytocannabinoid standards were prepared by diluting the stock solutions with ACN to get the final concentrations indicated in Table S1. Each dilution was run in triplicate and the calibration curves were built using both UV and MS data. Area of the peaks for each analyte was plotted against nominal concentration and the back-calculated concentration was checked to be within 15% of the nominal value. Samples of hemp inflorescence and HHC hashish were extracted using the method reported in the German Pharmacopoeia for the extraction of phytocannabinoids from cannabis inflorescence^24^. The extracts HHC-1 and HHC-2 from hemp inflorescence were analysed after 100 × dilution with mobile phase, while the extract HHC-3 from HHC hashish was 1000 × diluted. The sample of pure HHC (HHC-4) was injected at the concentration of 10 µg/mL obtained by dissolving 10 mg of the sample in 1 mL of ACN and preparing serial 10 × dilutions with mobile phase up to the desired concentration.

Quantification of cannabinoids was accomplished with both UV and MS data. The UV chromatograms were extracted at 228 nm. The exact *m/z* of the precursor ion in both HESI + and HESI- mode was extracted with a 5-ppm error from the HRMS TIC and used for calibration.

### Tetrad test

In the experiment, male C57BL6/J mice at 7 weeks old were used (*n* = 4). They were divided into two groups: one group received (9*R*)-HHC at a dose of 10 mg/kg dissolved in a vehicle (1:1:18; EtOH:Kolliphor EL:0.9% saline) via intraperitoneal (i.p.) administration, and the other group received only the vehicle as a control. The same animals were used for all four behavioural tests.

The effects of (9*R*)-HHC on hypomotility (measured using the open field test), hypothermia (measured by monitoring body temperature), antinociception (evaluated using the hot plate test), and catalepsy (assessed through the bar test) were assessed. These tests were performed following the procedures described by Metna-Laurent et al.^30^.

Statistical analysis of the data was conducted using the one-way analysis of variance (ANOVA) followed by Tukey’s multiple comparisons test.

*Body temperature.* The measurement of body temperature was performed after immobilizing the mouse. A probe was gently inserted 1 cm into the rectum, and the temperature was recorded once it stabilized. The probe was cleaned with 70% ethanol and dried with a paper towel between each mouse to prevent cross-contamination.

*Open field.* The open field test was conducted 30 min after administering the drug (or vehicle). The apparatus used for the test was cleaned with a 70% ethanol solution before each behavioural session. Naïve mice were randomly assigned to different treatment groups. The mice were placed in an open field arena (dimensions: length × width × height: 44 × 44 × 30 cm), and their ambulatory activity (total distance travelled in cm) was recorded and analyzed for a duration of 15 min. An automated behavioural tracking system called Any-maze, specifically the Video-tracking software by Ugo Basile, was used to record and analyze the behaviours.

*Bar test.* The bar test was conducted to assess catalepsy. A glass rod measuring 40 cm in length and 0.4 cm in diameter was horizontally elevated 5 cm above the surface. The mouse’s forelimbs were positioned on the bar while the hind legs remained on the floor of the cage, ensuring that the mouse was not lying down on the floor. The chronometer was started when the mouse held onto the bar with both forelimbs, and it was stopped when the mouse descended from the bar (i.e., when the two forepaws touched the floor) or after 5 min (cut-off time). Catalepsy was measured as the duration in seconds that the mouse held the elevated bar.

*Hot plate.* Each mouse was placed on a hot plate (Ugo Basile), which was kept at the constant temperature of 52 °C. Licking of the hind paws or jumping were considered as a nociceptive response (NR) and the latency was measured in s 85 min after drug or vehicle administration, taking a cut-off time of 30 s to prevent tissue damage. The hot plate test was performed to evaluate antinociceptive effects. Each mouse was placed on a hot plate set at a constant temperature of 52 °C. The licking of hind paws or jumping were considered nociceptive responses (NR), and the latency to respond was measured in seconds. The measurement was taken 85 min after drug or vehicle administration, with a cut-off time of 30 s to prevent tissue damage.

### Animals

In the described experiments, male C57BL/6 mice from Charles River (Italy) were used. The mice weighed between 18 and 20 g. The mice were acclimated to the laboratory conditions for at least 1 week before the start of the experiments. The laboratory maintained a 12-h light/dark cycle with the lights turning on at 6:00 A.M. The temperature in the facility was maintained at 20–22 °C, and the humidity was kept at 55–60%. The mice were housed in cages with three mice per cage. They had access to standard chow and tap water ad libitum, meaning they could eat and drink freely.

The experimental procedures conducted in this study received ethical approval from the Animal Ethics Committee of the University of Campania “L. Vanvitelli” in Naples, Italy. The specific protocol number for the experiments was 24/2023-PR. All the experiments were conducted in accordance with the regulations outlined by the Italian law (D.L. 116/92) and the European Commission (O.J. of E.C. L358/1, 18/12/86) regarding the protection of animals used for research purposes. The experimental methods described in the study also followed the ARRIVE guidelines, which provide recommendations for reporting animal research^31^. Animal care and welfare were the responsibility of trained personnel who adhered to the relevant Italian and European regulations. Every effort was made to minimize the number of animals used in the experiments and to prevent any unnecessary suffering or harm to the animals during the course of the study. (Fig. S1).

## Supplementary Information

Supplementary Information.

## Data Availability

All data generated or analysed during this study are included in this published article and its supplementary information files.
