# Supplementary material for: Synthesis and pharmacological activity of the epimers of hexahydrocannabinol (HHC)
Source: Sci Rep. 2023 Jul 8;13:11061. doi: 10.1038/s41598-023-38188-5 (PMC10329643; doi:10.1038/s41598-023-38188-5)
Supplement: Supplementary file 1 — Supplementary Information. [file 41598_2023_38188_MOESM1_ESM.docx]

**Synthesis and pharmacological activity of the epimers of hexahydrocannabinol (HHC)**

Fabiana Russo^a,b^, Maria Angela Vandelli^c^, Giuseppe Biagini^b^, Martin Schmid^d^, Livio Luongo^e^, Michela Perrone^e^, Federica Ricciardi^e^, Sabatino Maione^e^, Aldo Laganà^f^, Anna Laura Capriotti^f^, Alfonso Gallo^g^, Luigi Carbone^h^, Elisabetta Perrone^h^, Giuseppe Gigli^h^, Giuseppe Cannazza^c,h,^[[1]](#footnote-1)^*^, Cinzia Citti^h,c,*^

^a^ Clinical and Experimental Medicine PhD Program, University of Modena and Reggio Emilia, 41125 – Modena, Italy

^b^ Department of Biomedical, Metabolic and Neural Sciences, University of Modena and Reggio Emilia, 41125 – Modena, Italy

^c^ Department of Life Sciences, University of Modena and Reggio Emilia, Via Campi 103, 41125 – Modena, Italy

^d^ Institute of Pharmaceutical Sciences, Department of Pharmaceutical Chemistry, University of Graz, Schubertstraße 1, Graz, A-8010, Austria

^e^ Department of Experimental Medicine, Division of Pharmacology, Università della Campania “L. Vanvitelli”, Via Santa Maria di Costantinopoli 16, 80138, Naples, Italy.

^f^ Department of Chemistry, Sapienza University of Rome, Piazzale Aldo Moro 5, 00185 – Rome, Italy

^g^ Department of Chemistry, Istituto Zooprofilattico Sperimentale del Mezzogiorno, Via Salute 2, 80055 Portici, Italy

^h^ Institute of Nanotechnology – CNR NANOTEC, Campus Ecotekne, Via Monteroni, 73100 – Lecce, Italy

**Table of Content**

| **Table S1.** ^1^H NMR assignments (δ) of (9*S*)-HHC and (9*R*)-HHC | … SI-2 |
| --- | --- |
| **Figure S1.** ^1^H NMR spectroscopic characterization of (9*S*)-HHC | … SI-3 |
| **Figure S2.** ^13^C NMR spectroscopic characterization of (9*S*)-HHC | … SI-3 |
| **Figure S3.** ^1^H NMR spectroscopic characterization of (9*R*)-HHC | … SI-4 |
| **Figure S4.** ^13^C NMR spectroscopic characterization of (9*R*)-HHC | … SI-4 |
| **Figure S5.** HPLC-UV chromatograms of the analysed samples | … SI-5 |
| **Figure S6.** MS data of the unknown impurity in sample HHC-4 | … SI-6 |
| **Table S2.** Calibration parameters of the analytes | … SI-7 |

**Table S1.** ^1^H NMR assignments for (9S)-HHC and (9R)-HHC**.** In bold the discriminant signals.

|  | ^1^H chemical shifts (ppm) | |
| --- | --- | --- |
|  |  |  |
| Atom number | **(9*S*)-HHC** | **(9*R*)-HHC** |
| 2 | 6.07 | 6.07 |
| 4 | 6.25 | 6.25 |
| 6a | 1.46-1.44 | 1.46-1.44 |
| 7 | 1.68-1.61 | 1.87-1.80  1.16-1.07 |
| 8 | 1.68-1.61 | 1.87-1.80  1.16-1.07 |
| 9 | 2.11 | 1.67-1.60 |
| 10a | **2.67** | **2.49-2.37** |
| 10α | **2.92-2.79** | **3.03** |
| 10β | 1.33-1.28 | 0.78 |
| 11 | **1.13** | **0.94** |
| 12 | 1.36 | 1.36 |
| 13 | 1.09 | 1.06 |
| -OH | 4.61 | 4.64 |
| 1’ | 2.46-2.38 | 2.49-2.37 |
| 2’ | 1.58-1.52 | 1.59-1.52 |
| 3’ | 1.33-1.28 | 1.34-1.24 |
| 4’ | 1.33-1.28 | 1.34-1.24 |
| 5’ | 0.88 | 0.88 |

**Figure S1.** ^1^H NMR of (9S)-HHC

**Figure S2.** ^13^C NMR of (9S)-HHC

**Figure S3.** ^1^H NMR of (9R)-HHC

**Figure S4.** ^13^C NMR of (9S)-HHC


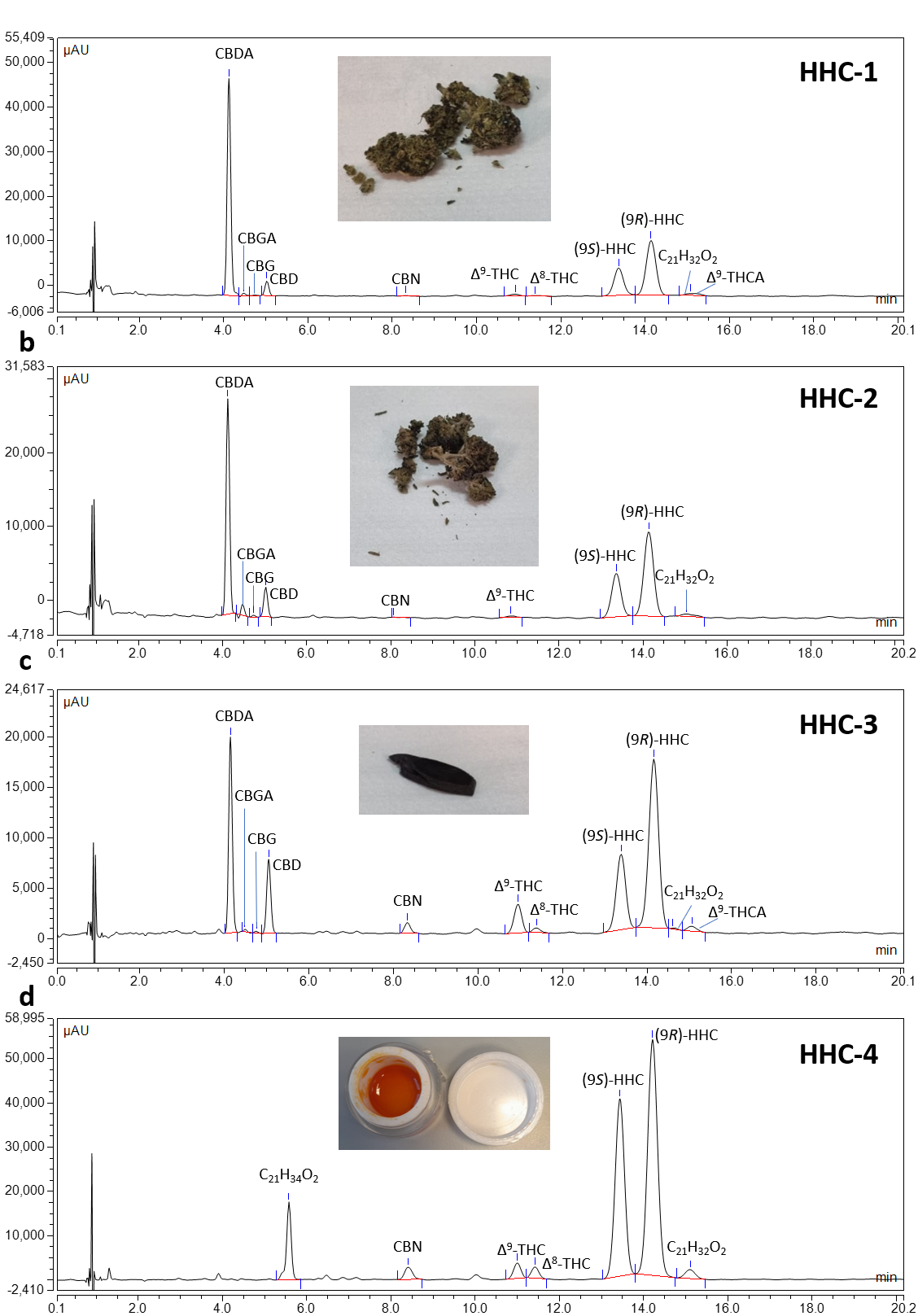


**Figure S5.** HPLC-UV chromatograms of the four samples: industrial hemp inflorescences (HHC-1 (**a**) and HHC-2 (**b**)), HHC hashish (HHC-3) (**c**) and pure HHC (HHC-4) (**d**). Picture of each sample is attached to the corresponding chromatogram.


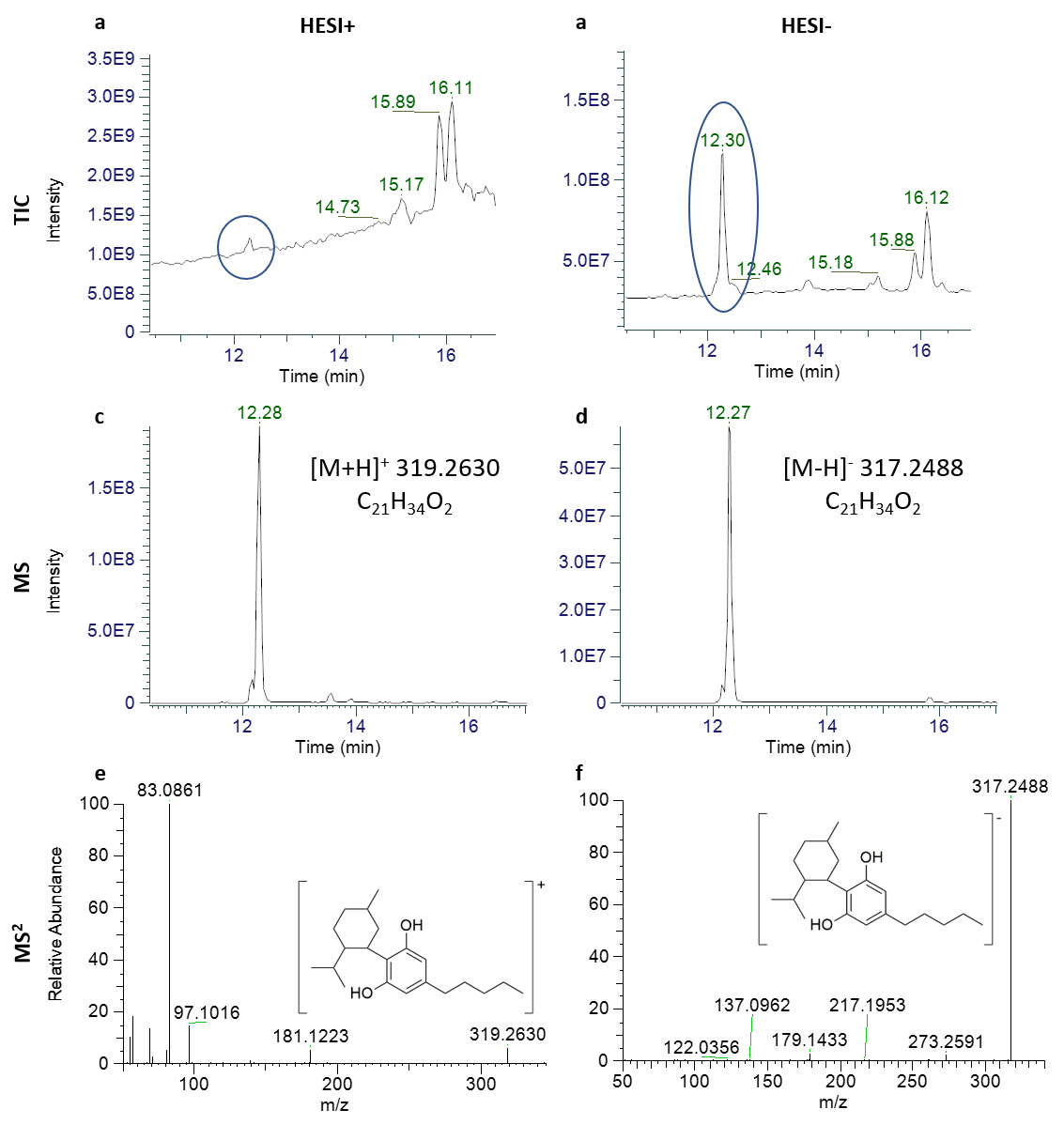


**Figure S6.** MS data of unknown impurity in sample HHC-4. Total ion current in HESI+ (**a**) and HESI- mode (**b**); extracted exact mass in HESI + (**c**) and HESI- mode (**d**); MS^2^ spectrum in HESI+ and HESI- mode (f).

**Table S2.** UV Calibration parameters (linearity range and coefficient of determination R^2^), retention time (R_T_) and MS precursor ions in HESI+ and HESI- mode of the analytes.

| Compound | *R_T_* (min) | [M+H]^+^ | [M-H]^-^ | Linearity range (µg/mL) | *R^2^* |
| --- | --- | --- | --- | --- | --- |
| CBDA | 11.60 | 359.2217 | 357.2071 | 0.1-10.0 | 0.99791 |
| CBGA | 12.10 | 361.2373 | 359.2228 | 0.1-10.0 | 0.99833 |
| CBG | 12.30 | 317.2475 | 315.2330 | 0.1-10.0 | 0.99939 |
| CBD | 12.42 | 315.2319 | 313.2173 | 0.1-10.0 | 0.99813 |
| CBN | 14.60 | 311.2006 | 309.1864 | 0.1-10.0 | 0.99951 |
| Δ^9^-THC | 15.73 | 315.2319 | 313.2173 | 0.1-10.0 | 0.99938 |
| Δ^8^-THC | 15.95 | 315.2319 | 313.2173 | 0.1-10.0 | 0.99917 |
| CBC | 16.55 | 315.2319 | 313.2173 | 0.1-10.0 | 0.99810 |
| (9*S*)-HHC | 16.59 | 317.2475 | 315.2330 | 1.0-20.0 | 0.99959 |
| (9*R*)-HHC | 16.88 | 317.2475 | 315.2330 | 1.0-20.0 | 0.99959 |
| Δ^9^-THCA | 17.20 | 359.2217 | 357.2071 | 0.1-10.0 | 0.99874 |
| CBCA | 17.90 | 359.2217 | 357.2071 | 0.1-10.0 | 0.99746 |

1. * Corresponding authors:

   Giuseppe Cannazza, Ph.D., Email: [giuseppe.cannazza@unimore.it](mailto:giuseppe.cannazza@unimore.it); Tel.: +39 059 2055013.; Fax: +39 059 2055750.

   Cinzia Citti, Ph.D., Email: [cinzia.citti@unimore.it](mailto:cinzia.citti@unimore.it); Tel.: +39 0832 319206. [↑](#footnote-ref-1)
